# Novel allele *elh* of the *UBP14* gene affects plant organ size via cell expansion in *Arabidopsis thaliana.*

**DOI:** 10.17912/micropub.biology.000401

**Published:** 2021-06-01

**Authors:** Rakesh David, Pei Qin Ng, Lisa M. Smith, Iain R. Searle

**Affiliations:** 1 School of Agriculture, Food and Wine, The University of Adelaide, Australia; 2 School of Biological Sciences, The University of Adelaide, Australia; 3 Department of Animal and Plant Sciences, The University of Sheffield, UK; 4 School of Biological Sciences, The University of Adelaide, Australia

## Abstract

Plant organ size control is an essential process of plant growth and development. The regulation of plant organ size involves a complicated network of genetic, molecular interactions, as well as the interplay of environmental factors. Here, we report a temperature-sensitive hypocotyl elongation EMS-generated mutant, hereby referred to as *elongated hypocotyl under high-temperature* (*elh*)*.* The elongated hypocotyl phenotype was prominent when the *elh* seedlings were grown at high temperature, 28^°^C, but not under the growth temperature of 21^°^C. We observed significantly larger organ sizes in *elh *plants, including cotyledons, petals and seeds. In *elh *plants, the cell sizes in cotyledons and petals were significantly larger than wild type. By measuring the cell density and organ area of cotyledons, petals and mature dissected embryos, we found no differences in total cell numbers in any organ indicating that cell expansion rather than cell proliferation was perturbed in *elh*. *elh *plants produced leaves at a slower rate than wild type plants, suggesting that perturbing the balance between cell division and cell expansion is linked to the developmental rate at which leaves are produced.

**Figure 1. The elh mutation affects cell and organ growth and is an allele of UBP14:**
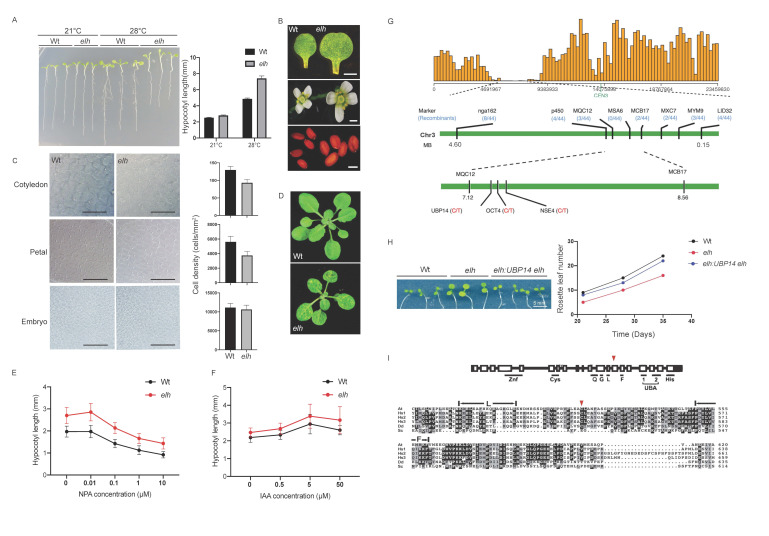
A. Hypocotyl elongation phenotypes of wild type and *elh Arabidopsis thaliana* seedlings grown at 21°C and 28°C. No hypocotyl length difference between wild type and *elh* seedlings was observed when grown at 21°C, however, the *elh* hypocotyls were significantly longer when grown at 28°C (Student’s t-test *p* < 0.05). B. Comparison of organ sizes (cotyledons, flowers, and seeds) of wild type and *elh*. C. Differential interference contrast (DIC) microscopy images showing cell sizes of cotyledons, petals and mature dissected embryos of wild type and *elh*. The cell density was calculated for each organ analysed. The cell densities between wild type and *elh* were significantly different in cotyledons and petals (*p* < 0.05) but not significantly different in embryos (Student’s t-test *p* > 0.05). D. Leaf growth of wild type and *elh* plants. The mutant produced fewer leaves than wild type after 16 days. Minor leaf photobleaching in *elh* is caused by the presence of an unlinked transgene (Smith **et al.*,* 2007) that does not affect leaf growth rate (Searle **et al.*,* 2010). The unlinked transgene was removed for subsequent experiments by backcrossing to wild type Col-0 three times and then self-pollinating twice. E. The effect of naphthylphthalamic acid (NPA) on hypocotyl length in wild type and *elh* at 28°C. F. Exogeneous auxin (IAA) effect on hypocotyl length in wild type and *elh* at 28°C. G. Bulk segregant analysis using a next generation mapping (NGM) approach mapped *elh* to chromosome 3. H. Transgene complementation of leaf growth rate in *elh* by using a wild type *UBP14* gene (Student’s t-test, *p* < 0.05). I. Multiple sequence alignments of UBP14 of *Arabidopsis thaliana* (*At*, AT3G20630), *Homo sapiens* (*Hs*, P54578), *Dictyostelium discoideum* (*Dd*, P54201), and *Saccharomyces cerevisiae* (*Sc*, P38237) adapted from Doeling *et al.* (2001). The red arrow indicates the site of the non-synonymous amino acid substitution in *elh* from cysteine to tyrosine. In panels A-H, the mean and standard deviation are shown (n= 20 plants).

## Description

Plant organ size control is an essential process of plant growth and development. The regulation of plant organ size involves a complicated network of genetic, molecular interactions, as well the interplay of environmental factors (Hepworth and Lenhlard, 2014). Two elements of plant organ size development are cell proliferation, resulting in increase in cell numbers, and cell expansion, which increases the overall organ size via larger cell area occupancy. However, our understanding of the fine-tuned regulation of organ size remains incomprehensive. Here, we report a temperature-sensitive hypocotyl elongation EMS-generated mutant, hereby referred to as *elongated hypocotyl under high-temperature* (*elh*)*.* The elongated hypocotyl phenotype is prominent when the *elh* seedlings are grown at high temperature, 28**^°^**C**,** but not under the growth temperature of21**^°^**C (Figure 1A). As hypocotyl elongation is driven primarily by cell elongation processes (Derbyshire *et al.* 2007), we compared the difference of organs size between in wild type and *elh* plants. We observed significantly larger organ sizes in *elh* plants, including cotyledons, petals and seeds when compared to the control (Student’s t-test *p* < 0.05, Figure 1B). We further inspected the cell size and number of these three organs in wild type and *elh* plants. In *elh*, the cell sizes in cotyledons and petals were significantly larger than wild type (Student’s t-test *p* < 0.05, Figure 1C). We also measured the cell density and organ area of cotyledons, petals and mature dissected embryos. By extrapolating cell numbers based on organ size, we found no differences in total cell numbers in either organ (Student’s t-test *p* > 0.05) indicating that cell expansion rather than cell proliferation was perturbed in *elh*. Plastochron, leaf initiation rate, has been previously linked to cell expansion (Wang *et al.* 2008). We observed reduced leaf number in *elh* plants when compared to wild type (Student’s t-test *p* < 0.05, Figure 1D) and a comprehensive analysis showed that *elh* plants produced leaves at a slower rate than wild type plants (Student’s t-test *p* < 0.05, Figure 1H), suggesting that perturbing the balance between cell division and cell expansion is linked to the developmental rate at which leaves are produced.

As auxin is a key hormone in regulating hypocotyl elongation and cell expansion (Gray *et al.* 1998; Chapman *et al.* 2012) and high auxin levels were shown to increase hypocotyl elongation (Wang *et al.* 2008; Zhao *et al.* 2001), we hypothesised that higher auxin levels in *elh* seedlings may cause the temperature-sensitive hypocotyl elongation phenotype. To test this hypothesis, we introduced the auxin inhibitor 1‐*N*‐naphthylphthalamic acid (NPA) into both wild-type and *elh* seedlings (Figure 1E). Higher levels of NPA reduced the hypocotyl length in both wild type and *elh* seedlings when grown at 28**^°^**C. At the concentration of 1 µM NPA, the length of *elh* hypocotyls were similar to wild type with no NPA (Figure 1 E). When we applied exogeneous auxin, indole-3-acetic acid (IAA) to wild type and *elh* seedlings (Figure 1F), the hypocotyl length elongation response at 28^°^C was similar in both genotypes (Figure 1F). Together these results suggest that *elh* accumulates higher auxin levels leading to a longer hypocotyl when compared to the wild type when grown at 28^°^C.

To understand the underlying molecular cause of the observed *elh* phenotypes, we performed bulk segregant analysis combined with Next Generation Mapping (NGM) to identify the causative mutation. Our NGM analysis suggested that the causative mutation in *elh* resulted in a non-synonymous amino acid substitution from cysteine to tyrosine in the *Ubiquitin carboxyl-terminal hydrolase 14* (*UBP14;* AT3G20630) gene (Figure 1G). *UBP14* genetically complemented both the cotyledon size and plastochron phenotypes in transgenic *elh* plants (Student’s t-test *p* < 0.05, Figure 1H) demonstrating that *UBP14* and *ELH* are the same gene. UBP14 is essential for early embryo development in *Arabidopsis thaliana* as null *ubp14* mutants are embryo lethal (Doelling *et al.* 2001)*.* Another *UBP14* allele has been shown to regulate organ size via regulating cell cycle kinases which affect both cell numbers and cell expansion (Xu *et al.* 2016). The *UBP14* allele, referred to as *da3*, has altered mRNA splicing resulting in a premature stop codon and the encoded protein lacks the conserved C-terminal His Box. Here, we characterized a novel *UBP14* allele gene that regulates cell organ size and elongation of hypocotyl under high temperature in *Arabidopsis*. Based on our findings, we postulate that UBP14regulates organ size via cell expansion through increased levels of auxin that affects the stability of HEAT SHOCK PROTEIN 90 (HSP90) dependent TIF1 co-receptor complexes. It has been shown that high temperatures lead to upregulation of auxin synthesis, followed by the activation of HSP90 to stabilize the TIR1 Auxin/IAA repressor degradation complex (Wang *et al.* 2016). The *elh* allele may act to destabilise the HSP90-TIR1 complex, leading to longer hypocotyls (Yamada *et al.*2009; Wang *et al.* 2016). Unlike the *da3* mutant allele that perturbs both cell expansion and proliferation (Xu *et al.* 2016), our findings suggest that these two functions can be separated by altering protein function. The *elh* allele identified here is potentially a useful tool to understand the role of UBP14 in plant development.

## Methods

**Plant materials and growth**

*Arabidopsis thaliana* (Columbia accession, Col-0) wild type and mutant plants were grown in Phoenix Biosystems chambers under metal halide lights as previously described in David *et al.* (2017). For plate experiments, seeds were first surface sterilized, plated on ½ MS medium supplemented with 1% sucrose and sealed as previously described in David *et al.* (2017). All plants were grown under long-day photoperiod conditions of 16 h light and 8 h darkness. For the temperature-sensitive hypocotyl length experiments, seedlings were grown at either 21**^°^**C or 28**^°^**C.

**Auxin inhibitor NPA and exogeneous auxin IAA treatments**

For NPA (Sigma Cat no- 33371-100MG) treatment, wild type and *elh* seeds were germinated on ½ MS media containing various concentration of NPA and along with control plates were transferred at 1 day after germination to 28**^°^**C. Seedlings were grown for 7 days and hypocotyl length was measured as described in Gray *et al.* (1998).

For IAA (Sigma Cat no-I-5148) treatment, seeds were plated on ½ MS media without IAA. Five days post germination, seedlings were transferred to plates containing different concentrations of IAA and allowed to grow for 2 days before hypocotyl length was measured as described in Chapman *et al.* (2012).

**Bulk segregant analysis and next-generation sequencing**

To perform a bulk segregant analysis approach, *elh* was crossed to a polymorphic *Landsberg erecta* (*Ler*) parent and in the F_2_ population, plants that had reduced plastochron were bulked together and sequenced on the Illumina Hiseq platform. The sequencing reads were trimmed using TrimGalore! (https://github.com/FelixKrueger/TrimGalore), trimmed reads were aligned to the TAIR10 reference genome using BWA (Li and Durbin 2010), followed by variant calling using SAMtools (Li *et al.* 2009). Identification of a SNP deserted region corresponding to *ELH* was achieved by plotting SNP frequencies across the 5 chromosomes using the Next Generation EMS mutation mapping tool (http://bar.utoronto.ca/ngm/cgi-bin/emap.cgi).

**Genetic complementation construct and generation of transgenic plants**

For the *ELH/UBP14* (AT3G20630) complementation construct, the full-length genomic sequence of *ELH/UBP14* including 2 kb upstream promoter sequence was PCR amplified and cloned into the Gateway cloning vector PCR8 TOPO-TA (Invitrogen). The respective insert was sequenced, then cloned into the destination vector pMDC100 via the Gateway Cloning approach (Curtis and Grossniklaus 2003). The construct was transformed into *elh* plants via *Agrobacterium tumefaciens*-mediated floral dipping as described by Davis *et al.* (2009). Transgenic plants were selected on kanamycin containing plates.
